# Supplementary material for: Unraveling a fine-scale high genetic heterogeneity and recent continental connections of an Arabian Peninsula population
Source: Eur J Hum Genet. 2021 Mar 22;30(3):307–19. doi: 10.1038/s41431-021-00861-6 (PMC8904638; doi:10.1038/s41431-021-00861-6)
Supplement: Supplementary file 1 — Supplementary Information [file 41431_2021_861_MOESM1_ESM.pdf]

Unraveling a fine-scale high genetic heterogeneity and recent continental connections of an Arabian Peninsula population

Muthukrishnan Eaaswarkhanth[1,*], Ajai K Pathak[2,3,*], Linda Ongaro[2,3], Francesco Montinaro[2,4], Prashantha Hebbar[1], Osama Alsmadi[1,5], Mait Metspalu[2], Fahd Al-Mulla[1], Thangavel Alphonse Thanaraj[1]

[1]*Department of Genetics and Bioinformatics, Dasman Diabetes Institute, P.O. Box 1180, Dasman 15462, Kuwait.*

[2]*Estonian Biocentre, Institute of Genomics, University of Tartu, Riia 23b, Tartu 51010, Estonia.*

[3]*Department of Evolutionary Biology, Institute of Molecular and Cell Biology, Riia 23b, Tartu 51010, Estonia.*

[4]*Department of Biology-Genetics, University of Bari, Bari, Italy.*

[5]*OA is currently affiliated with King Hussein Cancer Center, Amman, Jordan.*

[*]*These authors contributed equally to this work*

Corresponding Authors:

Thangavel Alphonse Thanaraj, Department of Genetics and Bioinformatics, Dasman Diabetes Institute, Kuwait, +965 2224 2999 Ext. 3320, alphonse.thangavel@dasmaninstitute.org.

Fahd Al-Mulla, Department of Genetics and Bioinformatics, Dasman Diabetes Institute, Kuwait, +965 2224 2999 Ext. 2211, fahd.almulla@dasmaninstitute.org.

Supplementary Text

Detailed Materials and methods section.

Supplementary Figures

Supplementary Fig. 1. (A) Geographical location of the populations included in the study. (B) Sample information, quality control measures and the population genetics analyses performed in the study.

Supplementary Fig. 2. The mean pairwise F_{ST} values showing genetic distance between individual populations.

Supplementary Fig. 3. Principal Component Analysis plot representing the first two principal components, PC1 and PC2, accounting for 2.11% and 1.72% of the variation respectively.

Supplementary Fig. 4. f_3 -statistics with Papuans as outgroup, comparing the genetic affinity of the Kuwaiti population subgroups to other modern populations.

Supplementary Fig. 5. Intra cluster Total Variation Distance for number and length of genomic fragments among the 40 clusters inferred by fineSTRUCTURE. (A) TVD estimated on number of fragments. (B) TVD estimated on length of fragments.

Supplementary Fig. 6. Intra cluster Total Variation Distance for number and length of genomic fragments among Kuwaiti individuals from the same cluster. Lower boxplots refer to TVD estimated on number of fragments and the upper boxplots refer to TVD estimated on length of fragments.

Supplementary Fig. 7. Ancestry proportions of the main eight Kuwait clusters as inferred by NNLS analysis using North/East Europe, Bedouins, Yoruba, Druze and North Africa clusters as putative sources.

Supplementary Fig. 8. Inferred admixture sources and times by GLOBETROTTER population based analysis. (A) The x and y axes show the minor and major sources, respectively, as inferred by GLOBETROTTER. (B) Type of admixture.

Supplementary Tables

Supplementary Table 1. Details of the populations included in the study.

Supplementary Table 2. f_4-statistics comparing the genetic affinity of the Kuwait population subgroups to other modern populations.

Supplementary Table 3. f_4-statistics for relative allele sharing of the Kuwait population subgroups to ancient West Eurasian individuals.

Supplementary Table 4. f_4-ratio estimates of Neanderthal ancestry in the Kuwait population.

Supplementary Table 5. Time of admixture estimate using ALDER.

Supplementary Text

Materials and methods

Samples

We included samples of 620 Kuwaiti individuals from the State of Kuwait who were part of a larger cohort collected for studies of metabolic disorders [1–3]. The participants were recruited through the following means: (i) from visitors to clinics and Open Day Events at our institute (ii) from visitors to our campaigns at malls, primary health centers and blood banks in each of the five governorates of the State of Kuwait, and (iii) from visitors to our campaigns at Kuwait University, where students are enrolled from all the governorates. All participants were healthy and enrolled after obtaining written informed consent. The study protocol was approved by the Ethical Review Committee of the Dasman Diabetes Institute, Kuwait. Participant recruitment, sample collection, and related procedures were conducted in accordance with the tenets of the Declaration of Helsinki, as detailed elsewhere [1–3]. Each of the 620 individuals were assigned to subgroups (Kuwait-P, Kuwait-S and Kuwait-B) based on their surnames that inform their respective ancestral lineage i.e., Persian, Saudi Arabian Tribe, and Bedouin. The surname lineage classification was described in detail in our previous study [1].

Genotyping and quality control

All the 620 individuals were genotyped using HumanOmniExpress arrays for 730,525 SNPs (Illumina, Inc., San Diego, CA, USA) in accordance with the manufacturers' protocol. Quality control checks and data filtering were conducted using the PLINK (version 1.9) whole genome data analysis toolset [4]. The dataset was filtered to contain only autosomal SNPs with a genotyping success rate of >90%, a minor allele frequency of >5%, and a probability (p) value of >0.001 as determined using the Hardy–Weinberg exact test. Only samples with a genotyping success rate of >90% were included for analysis. To ensure that the study individuals are unrelated, we examined relatedness among them using the "–genome" feature in PLINK with the threshold PI_HAT > 0.125 i.e., up to third degree relatives and randomly removed one sample per pair of related individuals. After QC and relatedness filtering, 583 individuals and 587,819 SNPs met the inclusion criteria for analysis (Supplementary Fig. 1). Genotype data has been deposited at the European Genome-phenome Archive (EGA), which is hosted by the European Bioinformatics

Institute (EBI) and the Centre for Genomic Regulation (CRG), under accession number EGAS00001005034.

Datasets, merging, and phasing

We merged our data with the published genome-wide SNP genotype data of global populations (Supplementary Fig. 1 and Supplementary Table 1) available from the Estonian Biocentre database (https://evolbio.ut.ee/). The combined dataset was filtered using PLINK version 1.9 [4] to include only autosomal SNPs with a minor allele frequency of >1%, a genotyping success rate of >97%, and only individuals with a genotyping success rate of >95%. The filtered combined dataset, which included 2139 individuals and 244,688 SNPs, was phased using the SHAPEIT algorithm [5] and used for further analyses. To avoid the effects of markers with a strong linkage disequilibrium, we thinned the marker set by removing SNPs with an r^2 value of >0.4 using a sliding window of 200 SNPs, shifted at intervals of 25 SNPs. The pruned dataset yielded 155,744 SNPs that were used for the relevant population genetics analyses, including Wright's *F*-statistic (F_{ST}), principal component analysis (PCA), the ADMIXTURE tool for ancestry estimation, and runs of homozygosity (RoH).

Population structure analyses: F_{ST}, PCA, ADMIXTURE, and RoH

To explore the population genetic structure, we initially computed the mean pairwise F_{ST} differences between all population groups using the Weir and Cockerham method [6] implemented in PLINK version 1.9 [4]. Next, we conducted PCA of the linkage disequilibrium-pruned combined dataset using the smartpca program included with the EIGENSOFT software package version 6.1.4 [7,8]. Further, we ran an unsupervised structure-like analysis using the ADMIXTURE tool (version 1.3.0) [9] on the same dataset 25 times at different time intervals, with K values ranging from 2 to 12. Notably, K = 9 was the best supported K value as determined from the lowest cross-validation indexes. RoH was estimated using PLINK version 1.9 [4] with a sliding window of 100 SNPs (1000 kb), allowing for one heterozygous and five missing calls per window.

Analyses to test admixture events and relative allele sharing: f_3 and f_4 statistics

We computed the f_3 and f_4 statistics using the *qp3Pop* and *qpDstat* programs (with f_4 mode: YES) implemented in the ADMIXTOOLS software package [10]. A dataset containing 244,688 SNPs

and 2139 individuals was used for analyses of the *f*-statistics of modern individuals. The dataset of modern genomes was merged with that of ancient specimens, which contained a combined total of 231,418 SNPs and 3697 individuals. To measure the genetic similarity of different Kuwaiti groups (i.e., modern and ancient), we computed the derived allele sharing of the Kuwaiti population using outgroup f_3 in the form of f_3(Mbuti; Pop1, X), where Pop1 is one of the three assigned groups of the Kuwaiti population, X is a modern or ancient West Eurasian population, and Mbuti is an outgroup (or Papuan to compare drift sharing between the Kuwait-B subgroup and other modern populations). Admixture f_3 was also calculated to infer the plausible admixing sources in the history of the Kuwaiti population determined by negative f_3 value with Z-score < -3 [10–12]. We calculated the f_4 statistic to evaluate the level of gene flow between contemporary populations of Kuwaitis and their regional neighbors and allele sharing between modern Kuwaitis and available published data of ancient individuals from surrounding regions. The f_4 statistic was applied to numerous population combinations in the form of f_4 (Pop1, Pop2, Pop3, Mbuti), where Pop1 and Pop2 are two Kuwaiti groups to be compared, while Pop3 is a modern or ancient population from a relevant region.

We estimated the amount of Neanderthal ancestry present in the Kuwaiti subgroups using the f_4-ratio implemented in the ADMIXTOOLS package [10]. We computed the Neanderthal fraction using the f_4-ratio in the form f_4 (Altai, Chimp, Target, Dinka) / f_4 (Altai, Chimp, Vindija, Dinka) following Petr et al. (2019) [13].

Haplotype-based fine-scale analyses: ChromoPainter and fineSTRUCTURE

We used the phased dataset with 244,688 SNPs and 2139 individuals for haplotype-based analyses. The ChromoPainter tool, designed to identify haplotypes in sequence data, was used to "paint" each individual as a combination of all other sequences [14]. As a first step, we estimated the -n (recombination scaling constant) and -M (per site mutation rate) parameters by running the software's EM option on a small subset of populations and five randomly selected chromosomes (3, 7, 10, 17, and 22), as described elsewhere [15]. The estimated values for the two parameters were -n = 510.86 and -M = 0.00033. Then, we executed the ChromoPainter tool in the "All vs All" mode (using the -a flag), where all individuals are considered as both donors and recipients.

Next, we analyzed the resulting painted dataset using the fineSTRUCTURE algorithm [14] to identify genetically homogenous clusters. We first ran the software performing 2 million burn-

in iterations and 4 million MCMC iterations thinned every 10,000. This generated an MCMC file (.xml) that we used to build the tree structure using the option --T 1.

The analyzed individuals were initially classified into 233 clusters, which we reduced to increase the interpretability of subsequent analyses. More specifically, we iteratively "climbed the tree," and the combined branches consisted of less than five clusters if at least one of them was composed of less than five individuals. The obtained tree was further refined by pooling together pairs or triplets of clusters if the pairwise total variation distance (TVD) based on the number of chunks shared among members of a branch was >0.035. After refinement, 40 clusters remained.

Non-negative least square (NNLS)

Starting from the copying vectors obtained with the ChromoPainter tool, we reconstructed the ancestry profile of each cluster or individual by applying a slight modification of the NNLS function of R software version 3.5.1 (https://www.r-project.org/), as described elsewhere [15,16]. Therefore, for each individual belonging to a Kuwait cluster and for each of these clusters, we decomposed their ancestry as a mixture (with proportions summing to 1) of five (North/East Europe, Bedouins1, Yoruba, Druze, and North Africa clusters) and three (only North/East Europe, Bedouins1, and Yoruba) putative ancestral sources.

Exploring the variability of Kuwaiti individuals via pairwise TVD analysis

To obtain a detailed picture of the variation underlying modern-day Kuwaiti population, we determined the pairwise TVD [16,17] among different individuals in specific clusters. TVD indicates the differences in ancestry profiles among individuals, where a high TVD value indicates high heterogeneity. First, we determined the TVD among individuals of the same cluster. Second, focusing only on Kuwaiti individuals, we determined the TVD among Kuwaitis and all the members of the respective clusters. Third, for each cluster, we determined the TVD only among Kuwaiti individuals. The analysis was performed in consideration of the number and length of genomic fragments inherited among individuals.

Estimation of admixture dates

The times of admixture events were investigated using the GLOBETROTTER software [18]. We applied an "individual" approach by analyzing each Kuwaiti individual alone [19]. First, we

estimated the time of the admixture event by applying the prop.ind=1, null.ind=0 approach to the 583 target individuals. Then, we performed 20 bootstrap iterations with the following settings: prop.ind=0, bootstrap.date.ind=1, and num.admixdates.bootstrap=1. For each of the inferred admixture events, we considered only those that were characterized by bootstrap values for the time of an admixture event between 1 and 400.

We also computed a weighted LD statistic estimating the date of admixture using ALDER version 1.03 that model the decay of admixture LD [20] to validate the admixture events calculated by ALDER and GLOBETROTTER are consistent.

References

1. Alsmadi O, Thareja G, Alkayal F, Rajagopalan R, John SE, Hebbar P, et al. Genetic substructure of Kuwaiti population reveals migration history. PLoS One. 2013;8: e74913.

2. Hebbar P, Elkum N, Alkayal F, John SE, Thanaraj TA, Alsmadi O. Genetic risk variants for metabolic traits in Arab populations. Sci Rep. 2017;7: 40988.

3. Hebbar P, Nizam R, Melhem M, Alkayal F, Elkum N, John SE, et al. Genome-wide association study identifies novel recessive genetic variants for high TGs in an Arab population. J Lipid Res. 2018;59: 1951–1966.

4. Chang CC, Chow CC, Tellier LC, Vattikuti S, Purcell SM, Lee JJ. Second-generation PLINK: rising to the challenge of larger and richer datasets. Gigascience. 2015;4: 7.

5. Delaneau O, Marchini J, Zagury J-F. A linear complexity phasing method for thousands of genomes. Nat Methods. 2011;9: 179–181.

6. Weir BS, Cockerham CC. ESTIMATING F-STATISTICS FOR THE ANALYSIS OF POPULATION STRUCTURE. Evolution. 1984;38: 1358–1370.

7. Patterson N, Price AL, Reich D. Population structure and eigen analysis. PLoS Genet. 2006;2: e190.

8. Price AL, Patterson NJ, Plenge RM, Weinblatt ME, Shadick NA, Reich D. Principal components analysis corrects for stratification in genome-wide association studies. Nat Genet. 2006;38: 904–909.

9. Alexander DH, Novembre J, Lange K. Fast model-based estimation of ancestry in unrelated individuals. Genome Res. 2009;19: 1655–1664.

10. Patterson N, Moorjani P, Luo Y, Mallick S, Rohland N, Zhan Y, et al. Ancient admixture in human history. Genetics. 2012;192: 1065–1093.

11. Pathak AK. Identifying admixture and genetic ancestry in human populations via genetic drift pattern. Polymorphism. 2020;4: 5–20.

12. Reich D, Thangaraj K, Patterson N, Price AL, Singh L. Reconstructing Indian population history. Nature. 2009;461: 489–494.

13. Petr M, Pääbo S, Kelso J, Vernot B. Limits of long-term selection against Neandertal introgression. Proc Natl Acad Sci U S A. 2019;116: 1639–1644.

14. Lawson DJ, Hellenthal G, Myers S, Falush D. Inference of population structure using dense haplotype data. PLoS Genet. 2012;8: e1002453.

15. Montinaro F, Busby GBJ, Pascali VL, Myers S, Hellenthal G, Capelli C. Unravelling the hidden ancestry of American admixed populations. Nat Commun. 2015;6: 6596.

16. Leslie S, Winney B, Hellenthal G, Davison D, Boumertit A, Day T, et al. The fine-scale genetic structure of the British population. Nature. 2015;519: 309–314.

17. Busby GBJ, Hellenthal G, Montinaro F, Tofanelli S, Bulayeva K, Rudan I, et al. The Role of Recent Admixture in Forming the Contemporary West Eurasian Genomic Landscape. Curr Biol. 2015;25: 2878.

18. Hellenthal G, Busby GBJ, Band G, Wilson JF, Capelli C, Falush D, et al. A genetic atlas of human admixture history. Science. 2014;343: 747–751.

19. Ongaro L, Scliar MO, Flores R, Raveane A, Marnetto D, Sarno S, et al. The Genomic Impact of European Colonization of the Americas. Curr Biol. 2019;29: 3974–3986.e4.

20. Loh P-R, Lipson M, Patterson N, Moorjani P, Pickrell JK, Reich D, et al. Inferring admixture histories of human populations using linkage disequilibrium. Genetics. 2013;193: 1233–1254.

Supplementary Fig. 1. (A) Geographical location of the populations included in the study. (B) Sample information, quality control measures and the population genetics analyses performed in the study.

A

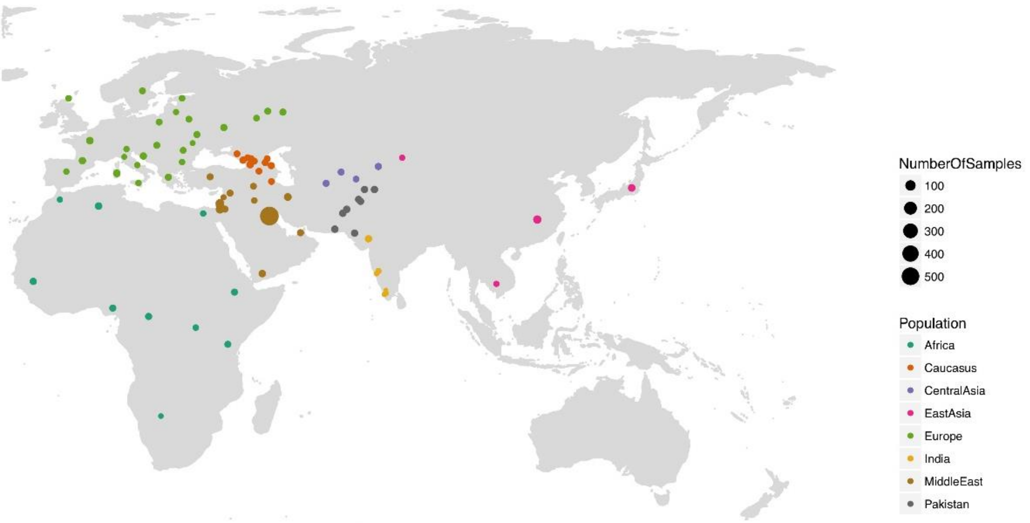

B

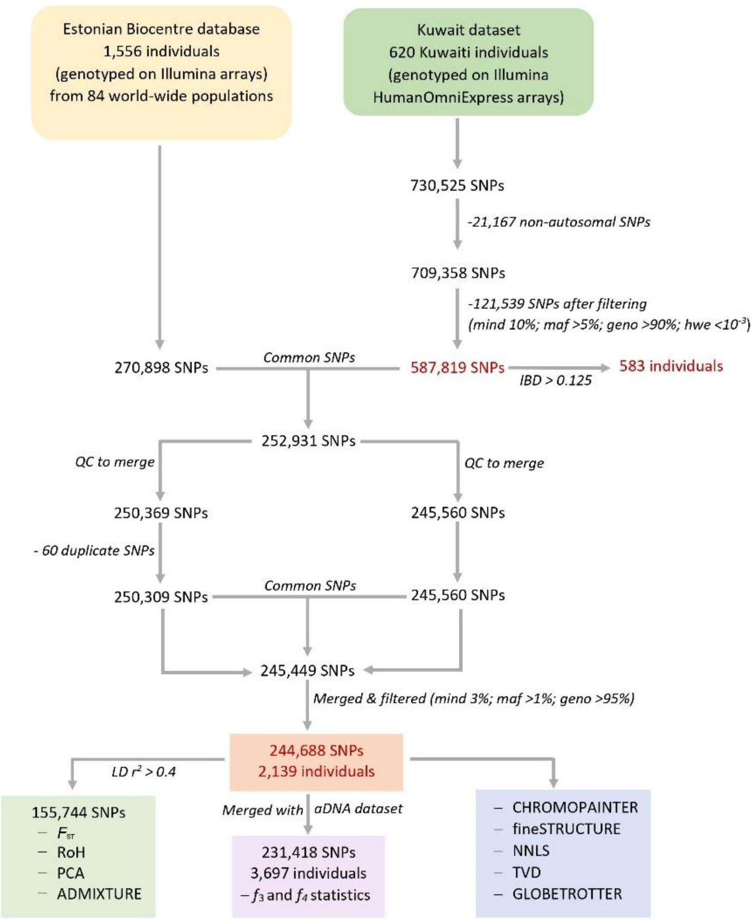

Supplementary Fig. 2. The mean pairwise F_{ST} values showing genetic distance between individual populations.

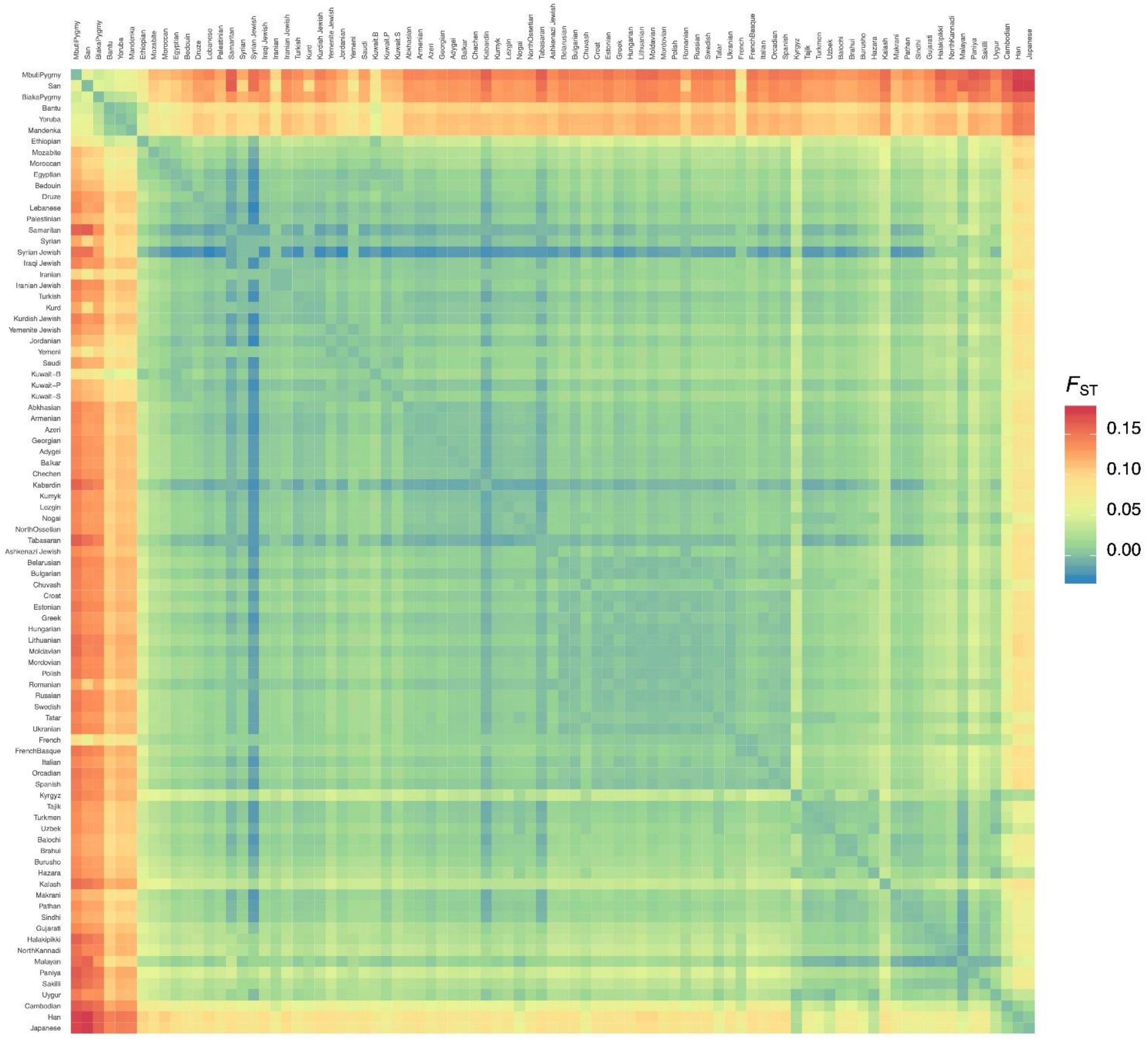

Supplementary Fig. 3. Principal Component Analysis plot based on allele frequency representing the first two principal components, PC1 and PC2, accounting for 2.11% and 1.72% of the variation respectively.

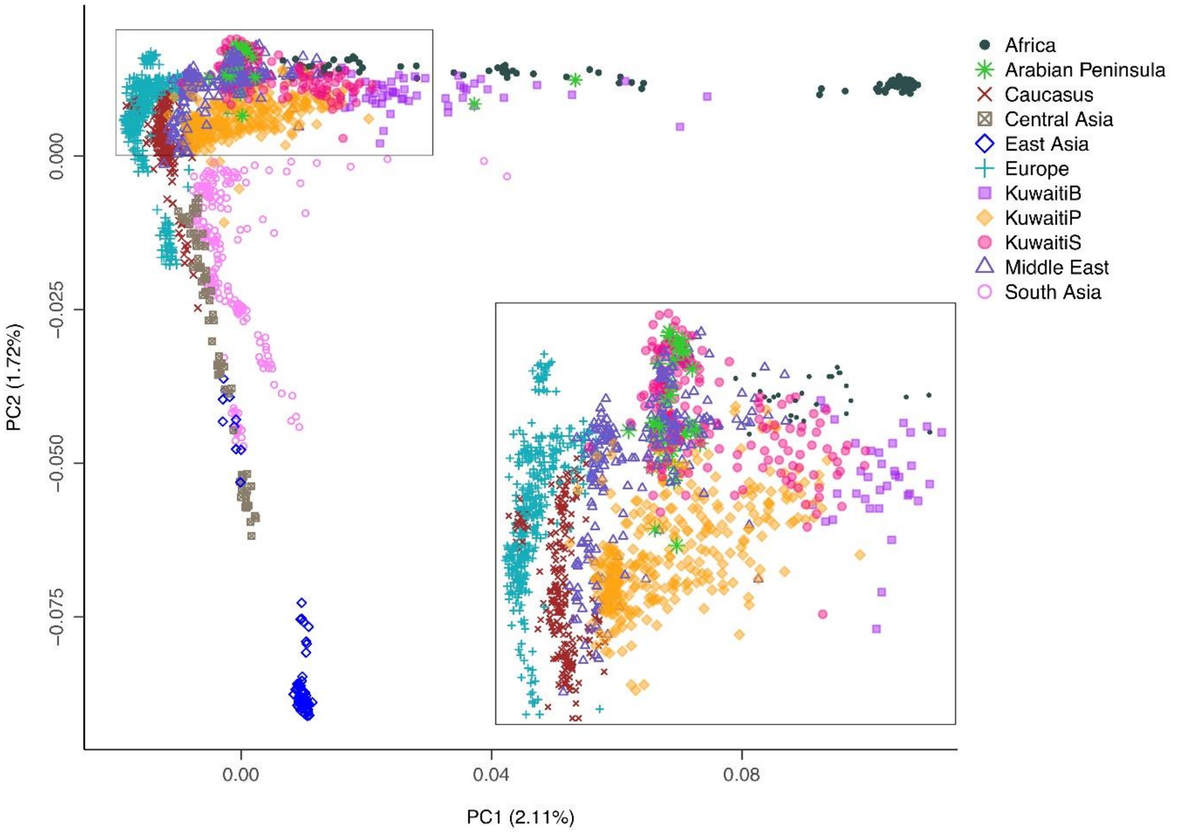

Supplementary Fig. 4. f_3 -statistics with Papuans as outgroup in the form D(Papauans; Pop1, X) where Pop1 represents a Kuwaiti subgroup and X is a present-day population, comparing the genetic affinity of the Kuwait population subgroups to other global modern populations.

The group color code for the populations on the y-axis: brown-Africa; blue-Arabian Peninsula; blue violet-Middle East; deep sky blue-Europe; coral-Caucasus; chartreuse-Central Asia; dark orange-South Asia.

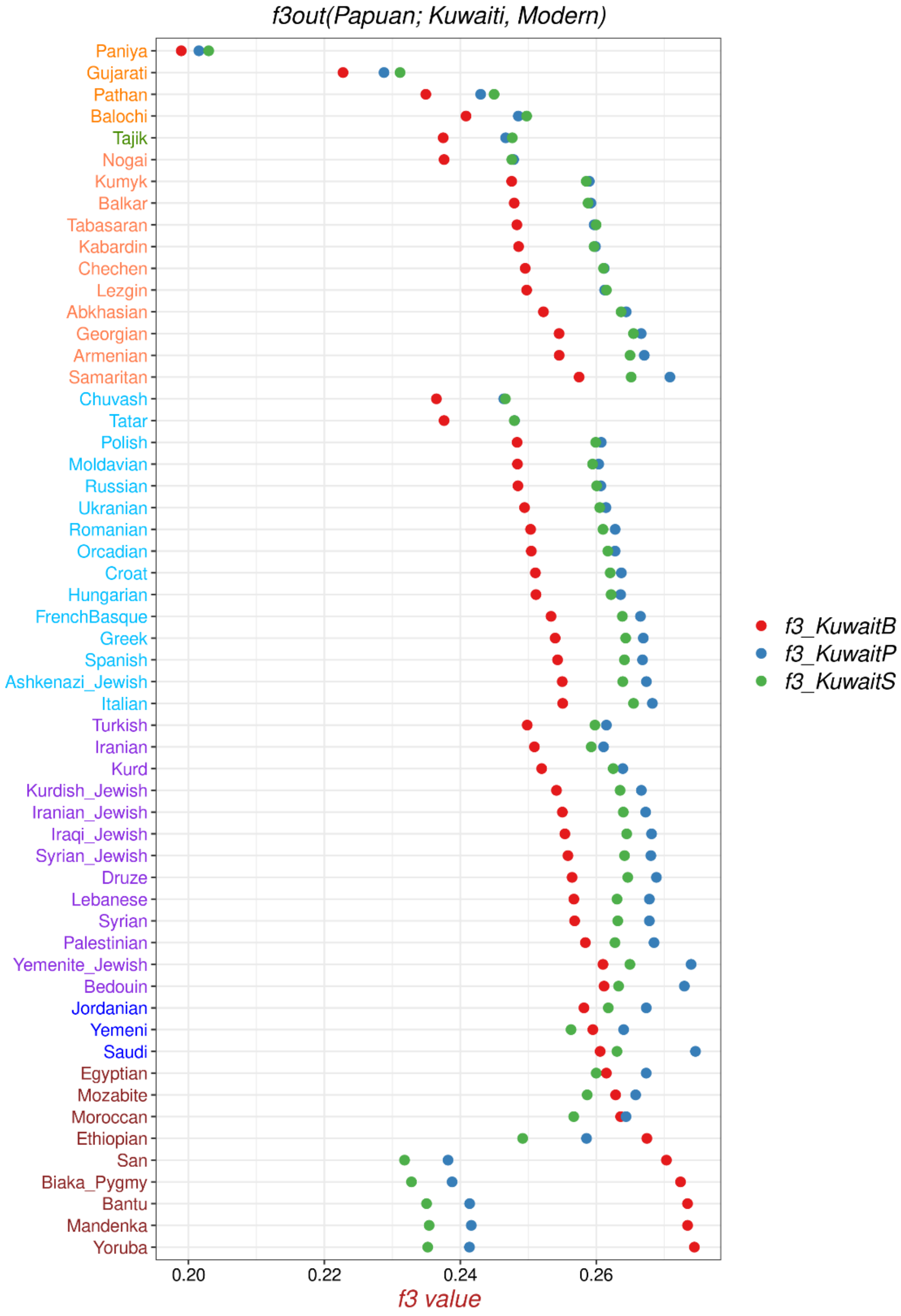

Supplementary Fig. 5. Intra cluster Total Variation Distance (TVD) for number and length of genomic fragments among the 40 clusters inferred by fineSTRUCTURE. (A) TVD estimated on number of fragments. (B) TVD estimated on length of fragments.

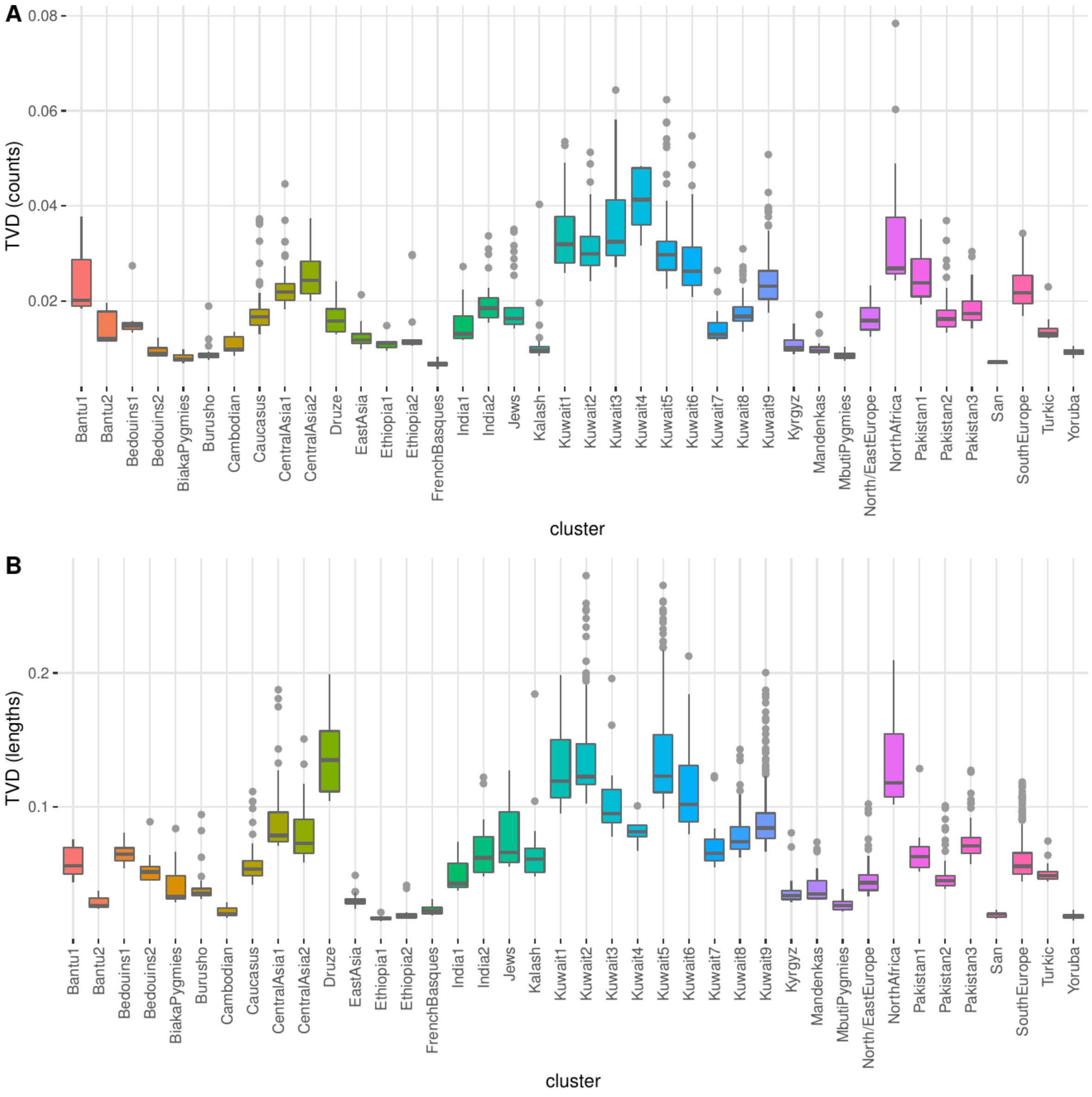

Supplementary Fig. 6. Intra cluster Total Variation Distance for number and length of genomic fragments among Kuwaiti individuals from the same cluster. Lower boxplots refer to TVD estimated on number of fragments, while upper boxplots refer to TVD estimated on length of fragments.

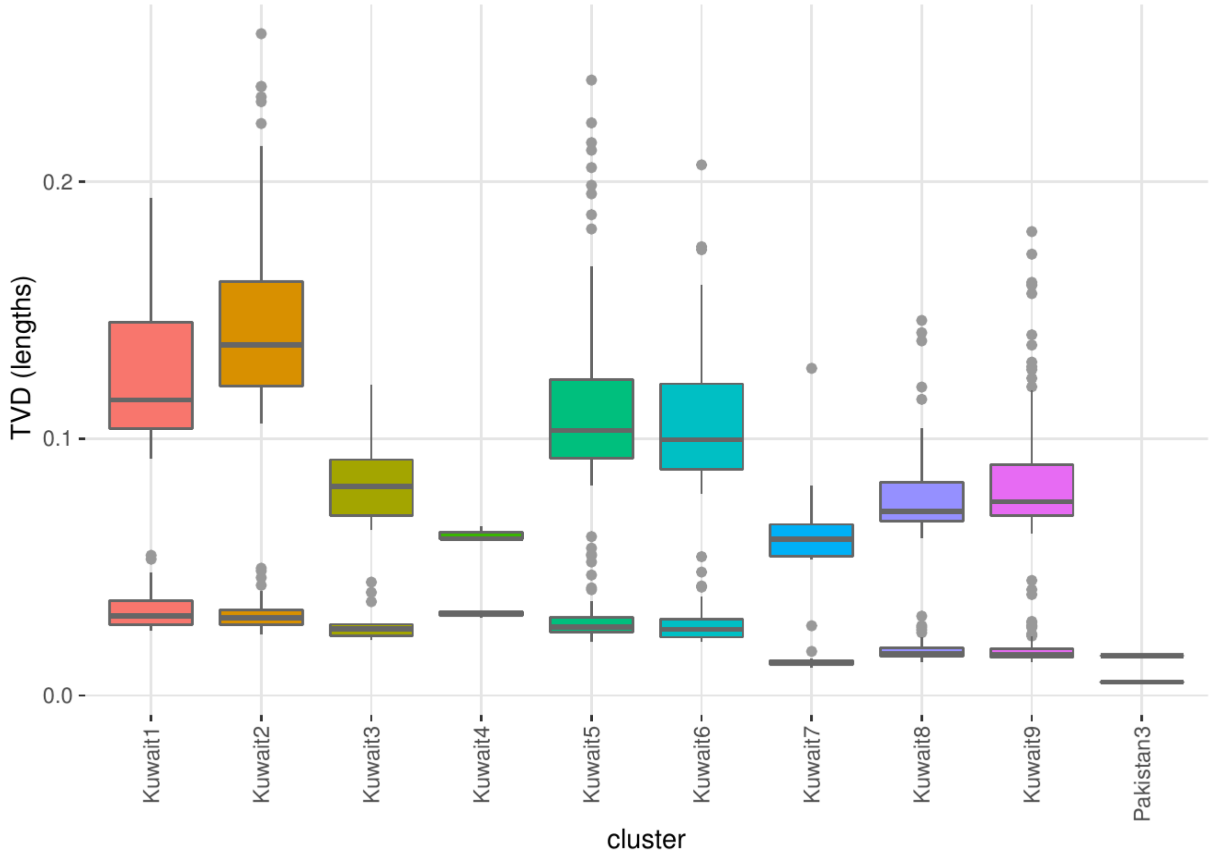

Supplementary Fig. 7. Ancestry proportions of the main eight Kuwait clusters as inferred by NNLS analysis using North/East Europe, Bedouins, Yoruba, Druze and North Africa clusters as putative sources.

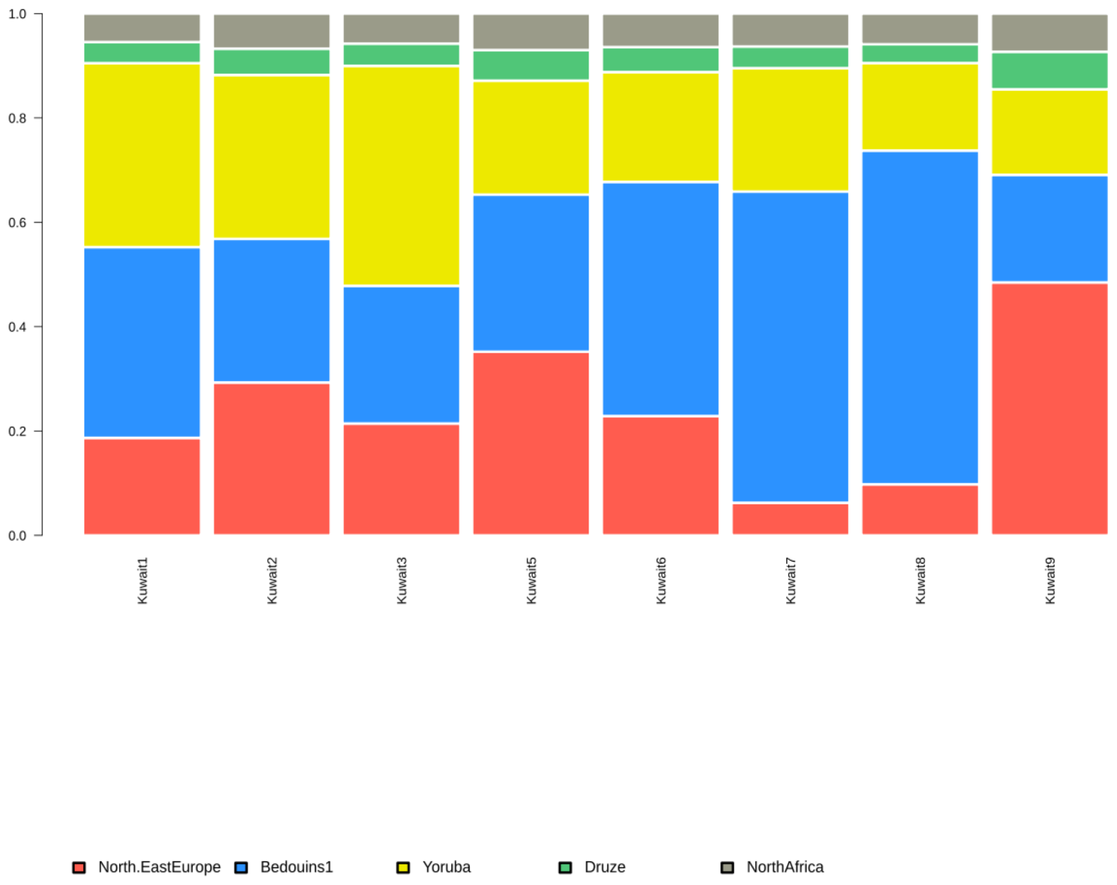

Supplementary Fig. 8. Inferred admixture sources by GLOBETROTTER population based analysis. (A) The x and y axes show the minor and major sources, respectively, as inferred by GLOBETROTTER. (B) Type of admixture.

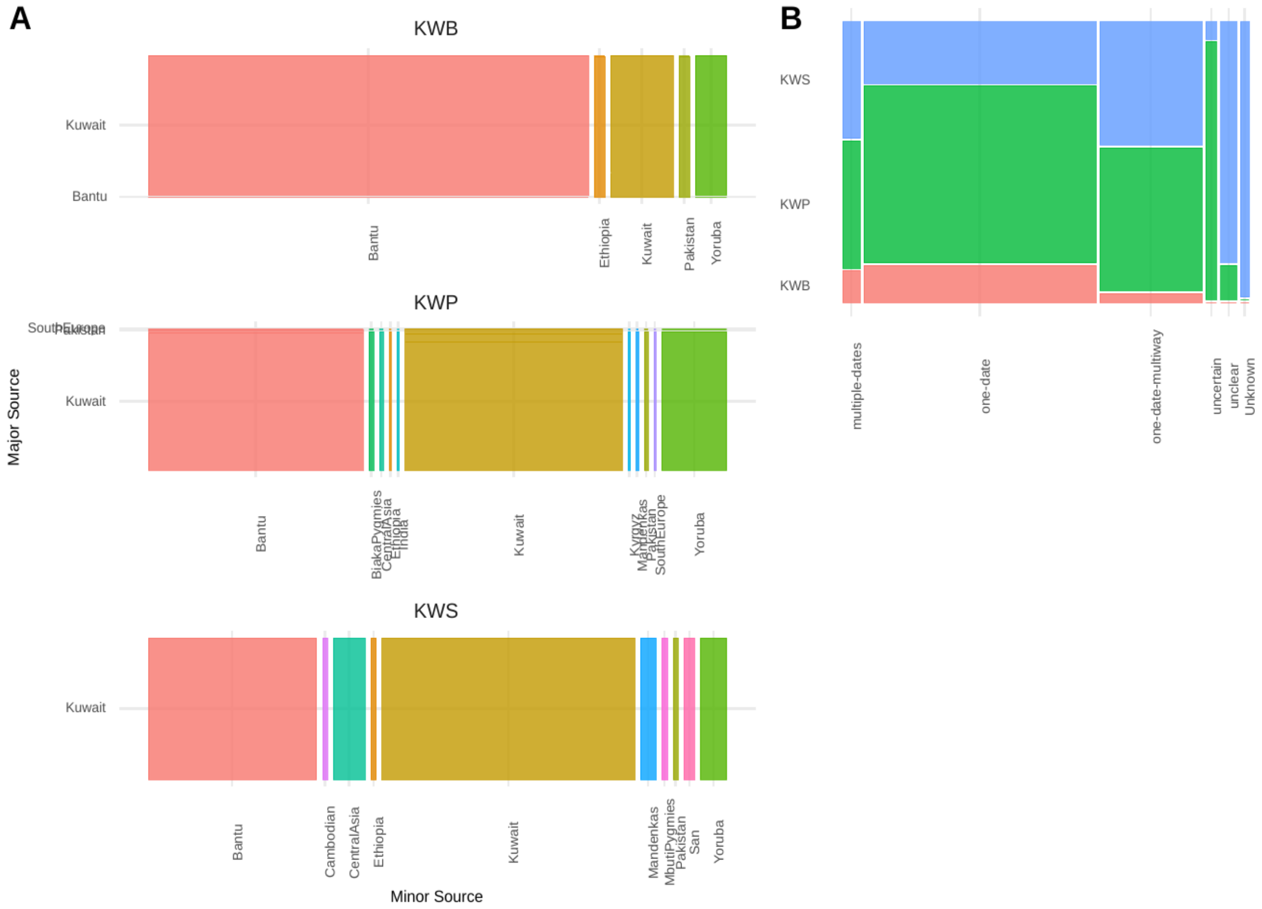

Supplementary Table 1. Details of the populations included in the study.

Region	Population	No. of individuals	Publication
Middle East	Bedouin	45	Li et al 2008
Middle East	Druze	45	Li et al 2008; Behar et al 2013
Middle East	Lebanese	8	Behar et al 2010
Middle East	Palestinian	52	Li et al 2008; Behar et al 2013
Middle East	Samaritan	2	Behar et al 2010
Middle East	Syrian	16	Behar et al 2010
Middle East	Turkish	19	Behar et al 2010
Middle East	Iranian	19	Behar et al 2010
Middle East	Kurd	6	Yunusbayev et al 2012
Middle East	Iranian Jewish	12	Behar et al 2010; Behar et al 2013
Middle East	Iraqi Jewish	13	Behar et al 2010; Behar et al 2013
Middle East	Kurdish Jewish	9	Behar et al. 2013
Middle East	Syrian Jewish	2	Behar et al. 2013
Middle East	Yemenite Jewish	18	Behar et al 2010; Behar et al 2013
Arabian Peninsula	Kuwait	583	Present Study
Arabian Peninsula	Saudi	20	Behar et al 2010
Arabian Peninsula	Yemeni	8	Behar et al 2010
Arabian Peninsula	Jordanian	20	Behar et al 2010
Sub-Saharan Africa	Bantu	18	Li et al 2008
Central Africa	Biaka Pygmy	22	Li et al 2008
North Africa	Egyptian	12	Behar et al 2010
North Africa	Ethiopian	19	Behar et al 2010
West Africa	Mandenka	22	Li et al 2008
Central Africa	Mbuti Pygmy	13	Li et al 2008
North Africa	Moroccan	10	Behar et al 2010
North Africa	Mozabite	27	Li et al 2008
South Africa	San	5	Li et al 2008
West Africa	Yoruba	21	Li et al 2008
South Caucasus	Abkhasian	23	Yunusbayev et al 2012; Behar et al 2013
South Caucasus	Armenian	16	Yunusbayev et al 2012
South Caucasus	Azeri	16	Yunusbayev et al 2015
South Caucasus	Georgian	30	Behar et al 2010; Behar et al 2013
North Caucasus	Adygei	17	Li et al 2008
North Caucasus	Balkar	22	Yunusbayev et al 2015; Yunusbayev et al 2012
North Caucasus	Chechen	20	Yunusbayev et al 2012
North Caucasus	Kabardin	3	Yunusbayev et al 2015
North Caucasus	Kumyk	17	Yunusbayev et al 2015; Yunusbayev et al 2012
North Caucasus	Lezgin	21	Behar et al 2010; Behar et al 2013
North Caucasus	Nogai	16	Yunusbayev et al 2012
North Caucasus	North Ossetian	18	Yunusbayev et al 2012; Behar et al 2013
North Caucasus	Tabasaran	3	Behar et al. 2013
Central Asia	Kyrgyz	20	Yunusbayev et al 2015
Central Asia	Tajik	15	Yunusbayev et al 2012
Central Asia	Turkmen	18	Yunusbayev et al 2012; Yunusbayev et al 2015
Central Asia	Uzbek	19	Behar et al 2010
East Asia	Cambodian	10	Li et al 2008

Region	Population	No. of individuals	Publication
East Asia	Han	44	Li et al 2008
East Asia	Japanese	28	Li et al 2008
East Asia	Uygur	10	Li et al 2008
Western Europe	French	28	Li et al 2008
Western Europe	French Basque	24	Li et al 2008
Western Europe	Italian	71	Li et al 2008; Behar et al 2013
Western Europe	Orcadian	15	Li et al 2008
Western Europe	Spanish	12	Behar et al 2010
Eastern Europe	Belarusian	17	Behar et al 2010; Behar et al 2013
Eastern Europe	Bulgarian	13	Yunusbayev et al 2012
Eastern Europe	Chuvash	19	Behar et al 2010; Yunusbayev et al 2015
Eastern Europe	Croat	24	Behar et al. 2013
Eastern Europe	Estonian	15	Raghavan et al. 2014
Eastern Europe	Greek	20	Behar et al. 2013
Eastern Europe	Hungarian	19	Behar et al 2010
Eastern Europe	Lithuanian	10	Behar et al 2010
Eastern Europe	Moldavian	7	Behar et al. 2013
Eastern Europe	Mordovian	15	Yunusbayev et al 2012
Eastern Europe	Polish	17	Behar et al. 2013
Eastern Europe	Romanian	16	Behar et al 2010
Eastern Europe	Russian	23	Yunusbayev et al 2015
Eastern Europe	Swedish	18	Behar et al. 2013
Eastern Europe	Tatar	20	Yunusbayev et al 2015
Eastern Europe	Ukranian	20	Yunusbayev et al 2012
Eastern Europe	Ashkenazi Jewish	29	Behar et al 2010; Behar et al 2013
South Asia (Pakistan)	Balochi	24	Li et al 2008
South Asia (Pakistan)	Brahui	25	Li et al 2008
South Asia (Pakistan)	Burusho	25	Li et al 2008
South Asia (Pakistan)	Hazara	22	Li et al 2008
South Asia (Pakistan)	Kalash	23	Li et al 2008
South Asia (Pakistan)	Makrani	25	Li et al 2008
South Asia (Pakistan)	Pathan	22	Li et al 2008
South Asia (Pakistan)	Sindhi	24	Li et al 2008
South Asia (India)	Gujarati	23	Hapmap3
South Asia (India)	Halakipikki	4	Metspalu et al 2011
South Asia (India)	Malayan	2	Behar et al 2010
South Asia (India)	North Kannadi	8	Behar et al 2010
South Asia (India)	Paniya	4	Behar et al 2010
South Asia (India)	Sakilli	4	Behar et al 2010

References

Behar DM, Metspalu M, Baran Y, Kopelman NM, Yunusbayev B, Gladstein A, et al. No evidence from genome-wide data of a Khazar origin for the Ashkenazi Jews. Hum Biol. 2013;85: 859–900.

Behar DM, Yunusbayev B, Metspalu M, Metspalu E, Rosset S, Parik J, et al. The genome-wide structure of the Jewish people. Nature. 2010;466: 238–242.

HapMap 3 Consortium I. Integrating common and rare genetic variation in diverse human populations. Nature. 2010. Available: https://www.ncbi.nlm.nih.gov/pmc/articles/pmc3173859/

Li JZ, Absher DM, Tang H, Southwick AM, Casto AM, Ramachandran S, et al. Worldwide human relationships inferred from genome-wide patterns of variation. Science. 2008;319: 1100–1104.

Metspalu M, Romero IG, Yunusbayev B, Chaubey G, Mallick CB, Hudjashov G, et al. Shared and unique components of human population structure and genome-wide signals of positive selection in South Asia. Am J Hum Genet. 2011;89: 731–744.

Raghavan M, Skoglund P, Graf KE, Metspalu M, Albrechtsen A, Moltke I, et al. Upper Palaeolithic Siberian genome reveals dual ancestry of Native Americans. Nature. 2014;505: 87–91.

Yunusbayev B, Metspalu M, Järve M, Kutuev I, Rootsi S, Metspalu E, et al. The Caucasus as an asymmetric semipermeable barrier to ancient human migrations. Mol Biol Evol. 2012;29: 359–365.

Yunusbayev B, Metspalu M, Metspalu E, Valeev A, Litvinov S, Valiev R, et al. The genetic legacy of the expansion of Turkic-speaking nomads across Eurasia. PLoS Genet. 2015;11: e1005068.

Supplementary Table 2. f_4-statistics comparing the genetic affinity of the Kuwait population subgroups to other modern populations.

Pop1	Pop2	Pop3	Pop4	D val	std err	Z val
Kuwait-P	Kuwait-S	Gujaratis	Mbuti_Pygmies	0.002097	0.000066	31.825
Kuwait-P	Kuwait-S	Gujjar	Mbuti_Pygmies	0.002043	0.000068	29.994
Kuwait-P	Kuwait-S	Kshatriya	Mbuti_Pygmies	0.002105	0.00007	29.985
Kuwait-P	Kuwait-S	Pathan	Mbuti_Pygmies	0.002001	0.000067	29.835
Kuwait-P	Kuwait-S	Ror	Mbuti_Pygmies	0.002052	0.000069	29.751
Kuwait-P	Kuwait-S	Chamar	Mbuti_Pygmies	0.002001	0.00007	28.733
Kuwait-P	Kuwait-S	Gond	Mbuti_Pygmies	0.001865	0.000067	27.647
Kuwait-P	Kuwait-S	Pamiris	Mbuti_Pygmies	0.001887	0.000068	27.564
Kuwait-P	Kuwait-S	Brahmins_UP	Mbuti_Pygmies	0.001982	0.000072	27.543
Kuwait-P	Kuwait-S	Irula	Mbuti_Pygmies	0.001898	0.00007	27.161
Kuwait-P	Kuwait-S	Balochi	Mbuti_Pygmies	0.001812	0.000067	27.065
Kuwait-P	Kuwait-S	Paniya	Mbuti_Pygmies	0.001864	0.000071	26.151
Kuwait-P	Kuwait-S	Ho	Mbuti_Pygmies	0.001778	0.000069	25.841
Kuwait-P	Kuwait-S	Chukchis	Mbuti_Pygmies	0.001838	0.000073	25.238
Kuwait-P	Kuwait-S	Tajiks	Mbuti_Pygmies	0.001747	0.000071	24.66
Kuwait-P	Kuwait-S	Karitiana	Mbuti_Pygmies	0.002152	0.000087	24.633
Kuwait-P	Kuwait-S	Pashtun	Mbuti_Pygmies	0.001816	0.000074	24.599
Kuwait-P	Kuwait-S	Kusundas	Mbuti_Pygmies	0.001787	0.000073	24.317
Kuwait-P	Kuwait-S	Kyrgyzians	Mbuti_Pygmies	0.001679	0.000071	23.675
Kuwait-P	Kuwait-S	Uygurs	Mbuti_Pygmies	0.001716	0.000073	23.505
Kuwait-P	Kuwait-S	Lezgins	Mbuti_Pygmies	0.001566	0.00007	22.221
Kuwait-P	Kuwait-S	Japanese	Mbuti_Pygmies	0.001652	0.000075	21.894
Kuwait-P	Kuwait-S	Han	Mbuti_Pygmies	0.001646	0.000075	21.887
Kuwait-P	Kuwait-S	Chuvash	Mbuti_Pygmies	0.001561	0.000072	21.551
Kuwait-P	Kuwait-S	Cambodians	Mbuti_Pygmies	0.001599	0.000075	21.312
Kuwait-P	Kuwait-S	Turkmens	Mbuti_Pygmies	0.001458	0.000069	21.236
Kuwait-P	Kuwait-S	Tatars	Mbuti_Pygmies	0.001496	0.000072	20.877
Kuwait-P	Kuwait-S	Chechens	Mbuti_Pygmies	0.001478	0.000071	20.7
Kuwait-P	Kuwait-S	Nogais	Mbuti_Pygmies	0.001434	0.000072	20.049
Kuwait-P	Kuwait-S	Kumyks	Mbuti_Pygmies	0.001391	0.00007	19.972
Kuwait-P	Kuwait-S	Parsis	Mbuti_Pygmies	0.001394	0.00007	19.863
Kuwait-P	Kuwait-S	Balkars	Mbuti_Pygmies	0.001398	0.00007	19.848
Kuwait-P	Kuwait-S	Onge	Mbuti_Pygmies	0.001538	0.000081	19.083
Kuwait-P	Kuwait-S	Tabasaran	Mbuti_Pygmies	0.001576	0.000084	18.711
Kuwait-P	Kuwait-S	Abkhasians	Mbuti_Pygmies	0.001314	0.00007	18.684
Kuwait-P	Kuwait-S	Kabardins	Mbuti_Pygmies	0.00145	0.00008	18.123
Kuwait-P	Kuwait-S	Ukranians	Mbuti_Pygmies	0.001271	0.000073	17.396
Kuwait-P	Kuwait-S	Polish	Mbuti_Pygmies	0.001297	0.000075	17.265
Kuwait-P	Kuwait-S	Russians	Mbuti_Pygmies	0.001347	0.000078	17.201
Kuwait-P	Kuwait-S	Georgians	Mbuti_Pygmies	0.001216	0.000073	16.596

Pop1	Pop2	Pop3	Pop4	D val	std err	Z val
Kuwait-P	Kuwait-S	Moldavians	Mbuti_Pygmies	0.001275	0.000077	16.582
Kuwait-P	Kuwait-S	Azeris	Mbuti_Pygmies	0.001153	0.00007	16.557
Kuwait-P	Kuwait-S	Orcadians	Mbuti_Pygmies	0.001231	0.000074	16.556
Kuwait-P	Kuwait-S	Hungarians	Mbuti_Pygmies	0.001148	0.000074	15.494
Kuwait-P	Kuwait-S	Turks	Mbuti_Pygmies	0.001076	0.00007	15.449
Kuwait-P	Kuwait-S	Kurds	Mbuti_Pygmies	0.001146	0.000075	15.254
Kuwait-P	Kuwait-S	Iranians	Mbuti_Pygmies	0.001045	0.000069	15.202
Kuwait-P	Kuwait-S	Croaitians	Mbuti_Pygmies	0.001088	0.000073	14.828
Kuwait-P	Kuwait-S	Romanians	Mbuti_Pygmies	0.00105	0.000073	14.387
Kuwait-P	Kuwait-S	Romanians	Mbuti_Pygmies	0.00105	0.000073	14.387
Kuwait-P	Kuwait-S	Armenians	Mbuti_Pygmies	0.000974	0.00007	14.008
Kuwait-P	Kuwait-S	Greeks	Mbuti_Pygmies	0.000848	0.000073	11.621
Kuwait-P	Kuwait-S	FrenchBasques	Mbuti_Pygmies	0.000831	0.000074	11.187
Kuwait-P	Kuwait-S	Spaniards	Mbuti_Pygmies	0.000832	0.000075	11.083
Kuwait-P	Kuwait-S	Tuscans	Mbuti_Pygmies	0.00081	0.000075	10.808
Kuwait-P	Kuwait-S	North_Italians	Mbuti_Pygmies	0.000803	0.000077	10.458
Kuwait-P	Kuwait-S	KurdishJews	Mbuti_Pygmies	0.000714	0.000072	9.893
Kuwait-P	Kuwait-S	IranianJewish	Mbuti_Pygmies	0.000674	0.00007	9.66
Kuwait-P	Kuwait-S	AshkenaziJewish	Mbuti_Pygmies	0.000625	0.00007	8.92
Kuwait-P	Kuwait-S	IraqiJewish	Mbuti_Pygmies	0.00058	0.000071	8.228
Kuwait-P	Kuwait-S	Druze	Mbuti_Pygmies	0.00044	0.000068	6.467
Kuwait-P	Kuwait-S	SyrianJews	Mbuti_Pygmies	0.000526	0.000091	5.752
Kuwait-P	Kuwait-S	Sardinians	Mbuti_Pygmies	0.000402	0.000073	5.5
Kuwait-P	Kuwait-S	Syrians	Mbuti_Pygmies	0.000331	0.00007	4.715
Kuwait-P	Kuwait-S	Lebanese	Mbuti_Pygmies	0.000302	0.000072	4.185
Kuwait-P	Kuwait-S	Jordanians	Mbuti_Pygmies	0.000093	0.000064	1.463
Kuwait-P	Kuwait-S	Palestinians	Mbuti_Pygmies	0.000054	0.000064	0.843
Kuwait-P	Kuwait-S	Samartians	Mbuti_Pygmies	0.000064	0.000091	0.708
**Kuwait-P**	**Kuwait-S**	**Mandenka**	**Mbuti_Pygmies**	**-0.00006**	**0.000035**	**-1.716**
**Kuwait-P**	**Kuwait-S**	**Mozabites**	**Mbuti_Pygmies**	**-0.000295**	**0.000061**	**-4.825**
**Kuwait-P**	**Kuwait-S**	**Egyptians**	**Mbuti_Pygmies**	**-0.000351**	**0.000064**	**-5.505**
**Kuwait-P**	**Kuwait-S**	**Yemenese**	**Mbuti_Pygmies**	**-0.000448**	**0.000063**	**-7.12**
**Kuwait-P**	**Kuwait-S**	**Moroccans**	**Mbuti_Pygmies**	**-0.000436**	**0.000061**	**-7.131**
**Kuwait-P**	**Kuwait-S**	**Kuwait-B**	**Mbuti_Pygmies**	**-0.000412**	**0.000051**	**-8.135**
**Kuwait-P**	**Kuwait-S**	**YemeniteJews**	**Mbuti_Pygmies**	**-0.000767**	**0.000068**	**-11.259**
**Kuwait-P**	**Kuwait-S**	**Bedouins**	**Mbuti_Pygmies**	**-0.000936**	**0.000065**	**-14.426**
**Kuwait-P**	**Kuwait-S**	**Ethiopians**	**Mbuti_Pygmies**	**-0.000865**	**0.000047**	**-18.392**
**Kuwait-P**	**Kuwait-S**	**Saudis**	**Mbuti_Pygmies**	**-0.001409**	**0.000067**	**-21.098**
Kuwait-B	Kuwait-S	Mandenka	Mbuti_Pygmies	-0.000501	0.000055	-9.063
Kuwait-B	Kuwait-S	Onge	Mbuti_Pygmies	-0.008888	0.000134	-66.54
Kuwait-B	Kuwait-S	Karitiana	Mbuti_Pygmies	-0.009863	0.000145	-67.986
Kuwait-B	Kuwait-S	Japanese	Mbuti_Pygmies	-0.00932	0.000136	-68.456
Kuwait-B	Kuwait-S	Cambodians	Mbuti_Pygmies	-0.009241	0.000132	-70.22

Pop1	Pop2	Pop3	Pop4	D val	std err	Z val
Kuwait-B	Kuwait-S	Han	Mbuti_Pygmies	-0.009316	0.000132	-70.68
Kuwait-B	Kuwait-S	Kusundas	Mbuti_Pygmies	-0.009414	0.000132	-71.079
Kuwait-B	Kuwait-S	Paniya	Mbuti_Pygmies	-0.009172	0.000127	-72.037
Kuwait-B	Kuwait-S	Ethiopians	Mbuti_Pygmies	-0.006282	0.000087	-72.054
Kuwait-B	Kuwait-S	Irula	Mbuti_Pygmies	-0.00942	0.000128	-73.339
Kuwait-B	Kuwait-S	Ho	Mbuti_Pygmies	-0.00919	0.000125	-73.757
Kuwait-B	Kuwait-S	Gond	Mbuti_Pygmies	-0.009306	0.000125	-74.349
Kuwait-B	Kuwait-S	Chamar	Mbuti_Pygmies	-0.009652	0.000129	-74.883
Kuwait-B	Kuwait-S	Chukchis	Mbuti_Pygmies	-0.009999	0.000133	-75.133
Kuwait-B	Kuwait-S	Kyrgyzians	Mbuti_Pygmies	-0.010047	0.000132	-76.383
Kuwait-B	Kuwait-S	Uygurs	Mbuti_Pygmies	-0.010231	0.000133	-76.971
Kuwait-B	Kuwait-S	Kshatriya	Mbuti_Pygmies	-0.010103	0.000131	-76.977
Kuwait-B	Kuwait-S	SyrianJews	Mbuti_Pygmies	-0.011594	0.00015	-77.081
Kuwait-B	Kuwait-S	Samartians	Mbuti_Pygmies	-0.011887	0.000152	-78.248
Kuwait-B	Kuwait-S	Moroccans	Mbuti_Pygmies	-0.008719	0.000111	-78.878
Kuwait-B	Kuwait-S	Brahmins_UP	Mbuti_Pygmies	-0.010208	0.000129	-79.085
Kuwait-B	Kuwait-S	Gujjar	Mbuti_Pygmies	-0.010316	0.00013	-79.283
Kuwait-B	Kuwait-S	Tabasaran	Mbuti_Pygmies	-0.011385	0.000143	-79.394
Kuwait-B	Kuwait-S	Yemenese	Mbuti_Pygmies	-0.00966	0.000121	-79.567
Kuwait-B	Kuwait-S	Kabardins	Mbuti_Pygmies	-0.011369	0.000142	-80.001
Kuwait-B	Kuwait-S	Gujaratis	Mbuti_Pygmies	-0.010037	0.000125	-80.145
Kuwait-B	Kuwait-S	Pashtun	Mbuti_Pygmies	-0.010743	0.000133	-80.662
Kuwait-B	Kuwait-S	Mozabites	Mbuti_Pygmies	-0.009262	0.000113	-81.633
Kuwait-B	Kuwait-S	Ror	Mbuti_Pygmies	-0.010548	0.000128	-82.26
Kuwait-B	Kuwait-S	Balochi	Mbuti_Pygmies	-0.010457	0.000127	-82.485
Kuwait-B	Kuwait-S	Chuvash	Mbuti_Pygmies	-0.011017	0.000133	-82.681
Kuwait-B	Kuwait-S	Tajiks	Mbuti_Pygmies	-0.010834	0.000131	-82.716
Kuwait-B	Kuwait-S	Nogais	Mbuti_Pygmies	-0.011097	0.000134	-82.963
Kuwait-B	Kuwait-S	Moldavians	Mbuti_Pygmies	-0.011531	0.000139	-83.083
Kuwait-B	Kuwait-S	Pathan	Mbuti_Pygmies	-0.010551	0.000127	-83.32
Kuwait-B	Kuwait-S	Parsis	Mbuti_Pygmies	-0.011009	0.000132	-83.586
Kuwait-B	Kuwait-S	Turkmens	Mbuti_Pygmies	-0.010855	0.00013	-83.641
Kuwait-B	Kuwait-S	Kurds	Mbuti_Pygmies	-0.011526	0.000137	-84.08
Kuwait-B	Kuwait-S	Tatars	Mbuti_Pygmies	-0.01113	0.000132	-84.224
Kuwait-B	Kuwait-S	Pamiris	Mbuti_Pygmies	-0.010906	0.000129	-84.264
Kuwait-B	Kuwait-S	Iranians	Mbuti_Pygmies	-0.011083	0.000131	-84.353
Kuwait-B	Kuwait-S	Polish	Mbuti_Pygmies	-0.011638	0.000138	-84.475
Kuwait-B	Kuwait-S	Tuscans	Mbuti_Pygmies	-0.011816	0.00014	-84.622
Kuwait-B	Kuwait-S	Russians	Mbuti_Pygmies	-0.011592	0.000137	-84.646
Kuwait-B	Kuwait-S	Egyptians	Mbuti_Pygmies	-0.009995	0.000118	-84.763
Kuwait-B	Kuwait-S	Balkars	Mbuti_Pygmies	-0.01136	0.000134	-84.843
Kuwait-B	Kuwait-S	Lezgins	Mbuti_Pygmies	-0.01141	0.000134	-85.027
Kuwait-B	Kuwait-S	KurdishJews	Mbuti_Pygmies	-0.011665	0.000137	-85.342

Pop1	Pop2	Pop3	Pop4	D val	std err	Z val
Kuwait-B	Kuwait-S	IranianJewish	Mbuti_Pygmies	-0.011606	0.000136	-85.412
Kuwait-B	Kuwait-S	Hungarians	Mbuti_Pygmies	-0.011657	0.000136	-85.432
Kuwait-B	Kuwait-S	Orcadians	Mbuti_Pygmies	-0.011627	0.000136	-85.464
Kuwait-B	Kuwait-S	Georgians	Mbuti_Pygmies	-0.011563	0.000135	-85.587
Kuwait-B	Kuwait-S	Chechens	Mbuti_Pygmies	-0.011447	0.000134	-85.632
Kuwait-B	Kuwait-S	Spaniards	Mbuti_Pygmies	-0.011667	0.000135	-86.285
Kuwait-B	Kuwait-S	FrenchBasques	Mbuti_Pygmies	-0.011834	0.000137	-86.288
Kuwait-B	Kuwait-S	Ukranians	Mbuti_Pygmies	-0.011537	0.000134	-86.288
Kuwait-B	Kuwait-S	Kumyks	Mbuti_Pygmies	-0.011396	0.000132	-86.34
Kuwait-B	Kuwait-S	Turks	Mbuti_Pygmies	-0.011456	0.000133	-86.435
Kuwait-B	Kuwait-S	Croaitians	Mbuti_Pygmies	-0.011706	0.000135	-86.464
Kuwait-B	Kuwait-S	Abkhasians	Mbuti_Pygmies	-0.011587	0.000134	-86.698
Kuwait-B	Kuwait-S	Jordanians	Mbuti_Pygmies	-0.010831	0.000125	-86.718
Kuwait-B	Kuwait-S	Lebanese	Mbuti_Pygmies	-0.011318	0.00013	-86.741
Kuwait-B	Kuwait-S	Azeris	Mbuti_Pygmies	-0.011366	0.000131	-86.891
Kuwait-B	Kuwait-S	Greeks	Mbuti_Pygmies	-0.011785	0.000135	-87.056
Kuwait-B	Kuwait-S	Romanians	Mbuti_Pygmies	-0.011655	0.000134	-87.08
Kuwait-B	Kuwait-S	Romanians	Mbuti_Pygmies	-0.011655	0.000134	-87.08
Kuwait-B	Kuwait-S	North_Italians	Mbuti_Pygmies	-0.011843	0.000136	-87.374
Kuwait-B	Kuwait-S	Syrians	Mbuti_Pygmies	-0.011289	0.000128	-88.09
Kuwait-B	Kuwait-S	IraqiJewish	Mbuti_Pygmies	-0.011731	0.000133	-88.443
Kuwait-B	Kuwait-S	Armenians	Mbuti_Pygmies	-0.011674	0.000132	-88.492
Kuwait-B	Kuwait-S	Sardinians	Mbuti_Pygmies	-0.011996	0.000135	-88.766
Kuwait-B	Kuwait-S	Palestinians	Mbuti_Pygmies	-0.011064	0.000125	-88.795
Kuwait-B	Kuwait-S	AshkenaziJewish	Mbuti_Pygmies	-0.011639	0.00013	-89.375
Kuwait-B	Kuwait-S	Druze	Mbuti_Pygmies	-0.011646	0.00013	-89.611
Kuwait-B	Kuwait-S	YemeniteJews	Mbuti_Pygmies	-0.011786	0.000128	-92.173
Kuwait-B	Kuwait-S	Bedouins	Mbuti_Pygmies	-0.011496	0.000123	-93.306
Kuwait-B	Kuwait-S	Saudis	Mbuti_Pygmies	-0.012053	0.000128	-93.91
Kuwait-B	Kuwait-P	Mandenka	Mbuti_Pygmies	-0.000441	0.000055	-8.064
Kuwait-B	Kuwait-P	Ethiopians	Mbuti_Pygmies	-0.005417	0.000085	-63.614
Kuwait-B	Kuwait-P	Moroccans	Mbuti_Pygmies	-0.008283	0.000113	-73.46
Kuwait-B	Kuwait-P	Onge	Mbuti_Pygmies	-0.010426	0.000142	-73.638
Kuwait-B	Kuwait-P	Yemenese	Mbuti_Pygmies	-0.009212	0.000123	-74.944
Kuwait-B	Kuwait-P	Samartians	Mbuti_Pygmies	-0.011951	0.000156	-76.491
Kuwait-B	Kuwait-P	Karitiana	Mbuti_Pygmies	-0.012015	0.000157	-76.655
Kuwait-B	Kuwait-P	Japanese	Mbuti_Pygmies	-0.010972	0.000142	-77.11
Kuwait-B	Kuwait-P	Mozabites	Mbuti_Pygmies	-0.008967	0.000116	-77.333
Kuwait-B	Kuwait-P	Cambodians	Mbuti_Pygmies	-0.01084	0.000139	-78.224
Kuwait-B	Kuwait-P	Han	Mbuti_Pygmies	-0.010962	0.00014	-78.328
Kuwait-B	Kuwait-P	SyrianJews	Mbuti_Pygmies	-0.01212	0.000154	-78.912
Kuwait-B	Kuwait-P	Egyptians	Mbuti_Pygmies	-0.009644	0.000119	-80.845
Kuwait-B	Kuwait-P	Kusundas	Mbuti_Pygmies	-0.011201	0.000139	-80.861

Pop1	Pop2	Pop3	Pop4	D val	std err	Z val
Kuwait-B	Kuwait-P	Paniya	Mbuti_Pygmies	-0.011036	0.000135	-81.863
Kuwait-B	Kuwait-P	Saudis	Mbuti_Pygmies	-0.010644	0.00013	-82.119
Kuwait-B	Kuwait-P	Ho	Mbuti_Pygmies	-0.010967	0.000133	-82.425
Kuwait-B	Kuwait-P	Kyrgyzians	Mbuti_Pygmies	-0.011725	0.000139	-84.084
Kuwait-B	Kuwait-P	Chukchis	Mbuti_Pygmies	-0.011837	0.000141	-84.129
Kuwait-B	Kuwait-P	Irula	Mbuti_Pygmies	-0.011318	0.000134	-84.25
Kuwait-B	Kuwait-P	Gond	Mbuti_Pygmies	-0.011172	0.000132	-84.351
Kuwait-B	Kuwait-P	Bedouins	Mbuti_Pygmies	-0.01056	0.000125	-84.816
Kuwait-B	Kuwait-P	YemeniteJews	Mbuti_Pygmies	-0.011019	0.000129	-85.607
Kuwait-B	Kuwait-P	Chamar	Mbuti_Pygmies	-0.011653	0.000136	-85.705
Kuwait-B	Kuwait-P	Jordanians	Mbuti_Pygmies	-0.010924	0.000127	-85.853
Kuwait-B	Kuwait-P	Uygurs	Mbuti_Pygmies	-0.011947	0.000139	-86.058
Kuwait-B	Kuwait-P	Kabardins	Mbuti_Pygmies	-0.012819	0.000149	-86.089
Kuwait-B	Kuwait-P	Palestinians	Mbuti_Pygmies	-0.011118	0.000128	-86.667
Kuwait-B	Kuwait-P	Tuscans	Mbuti_Pygmies	-0.012625	0.000145	-87.229
Kuwait-B	Kuwait-P	Lebanese	Mbuti_Pygmies	-0.01162	0.000133	-87.458
Kuwait-B	Kuwait-P	Tabasaran	Mbuti_Pygmies	-0.012961	0.000148	-87.782
Kuwait-B	Kuwait-P	IranianJewish	Mbuti_Pygmies	-0.01228	0.00014	-87.816
Kuwait-B	Kuwait-P	Brahmins_UP	Mbuti_Pygmies	-0.01219	0.000139	-88.003
Kuwait-B	Kuwait-P	Kshatriya	Mbuti_Pygmies	-0.012208	0.000139	-88.097
Kuwait-B	Kuwait-P	Kurds	Mbuti_Pygmies	-0.012672	0.000144	-88.109
Kuwait-B	Kuwait-P	Pashtun	Mbuti_Pygmies	-0.012559	0.000142	-88.154
Kuwait-B	Kuwait-P	KurdishJews	Mbuti_Pygmies	-0.012379	0.00014	-88.242
Kuwait-B	Kuwait-P	Syrians	Mbuti_Pygmies	-0.01162	0.000132	-88.28
Kuwait-B	Kuwait-P	Moldavians	Mbuti_Pygmies	-0.012807	0.000145	-88.384
Kuwait-B	Kuwait-P	Sardinians	Mbuti_Pygmies	-0.012397	0.00014	-88.645
Kuwait-B	Kuwait-P	Nogais	Mbuti_Pygmies	-0.012531	0.000141	-88.856
Kuwait-B	Kuwait-P	Spaniards	Mbuti_Pygmies	-0.012499	0.00014	-89.173
Kuwait-B	Kuwait-P	FrenchBasques	Mbuti_Pygmies	-0.012665	0.000142	-89.185
Kuwait-B	Kuwait-P	Polish	Mbuti_Pygmies	-0.012935	0.000145	-89.228
Kuwait-B	Kuwait-P	IraqiJewish	Mbuti_Pygmies	-0.012311	0.000137	-89.533
Kuwait-B	Kuwait-P	Iranians	Mbuti_Pygmies	-0.012128	0.000135	-89.571
Kuwait-B	Kuwait-P	Orcadians	Mbuti_Pygmies	-0.012858	0.000143	-89.712
Kuwait-B	Kuwait-P	Druze	Mbuti_Pygmies	-0.012086	0.000135	-89.723
Kuwait-B	Kuwait-P	Greeks	Mbuti_Pygmies	-0.012634	0.000141	-89.832
Kuwait-B	Kuwait-P	Gujjar	Mbuti_Pygmies	-0.012359	0.000138	-89.881
Kuwait-B	Kuwait-P	Chuvash	Mbuti_Pygmies	-0.012578	0.00014	-89.936
Kuwait-B	Kuwait-P	Parsis	Mbuti_Pygmies	-0.012404	0.000138	-90.113
Kuwait-B	Kuwait-P	Russians	Mbuti_Pygmies	-0.01294	0.000143	-90.285
Kuwait-B	Kuwait-P	Romanians	Mbuti_Pygmies	-0.012705	0.00014	-90.436
Kuwait-B	Kuwait-P	Romanians	Mbuti_Pygmies	-0.012705	0.00014	-90.436
Kuwait-B	Kuwait-P	Hungarians	Mbuti_Pygmies	-0.012805	0.000141	-90.503
Kuwait-B	Kuwait-P	Turkmens	Mbuti_Pygmies	-0.012312	0.000136	-90.514

Pop1	Pop2	Pop3	Pop4	D val	std err	Z val
Kuwait-B	Kuwait-P	North_Italians	Mbuti_Pygmies	-0.012647	0.000139	-90.69
Kuwait-B	Kuwait-P	Tajiks	Mbuti_Pygmies	-0.012581	0.000138	-90.864
Kuwait-B	Kuwait-P	Tatars	Mbuti_Pygmies	-0.012625	0.000139	-90.888
Kuwait-B	Kuwait-P	Croaitians	Mbuti_Pygmies	-0.012793	0.000141	-90.915
Kuwait-B	Kuwait-P	AshkenaziJewish	Mbuti_Pygmies	-0.012264	0.000135	-90.989
Kuwait-B	Kuwait-P	Azeris	Mbuti_Pygmies	-0.012519	0.000137	-91.123
Kuwait-B	Kuwait-P	Turks	Mbuti_Pygmies	-0.012532	0.000137	-91.164
Kuwait-B	Kuwait-P	Georgians	Mbuti_Pygmies	-0.012779	0.00014	-91.253
Kuwait-B	Kuwait-P	Ukranians	Mbuti_Pygmies	-0.012808	0.00014	-91.38
Kuwait-B	Kuwait-P	Gujaratis	Mbuti_Pygmies	-0.012134	0.000133	-91.409
Kuwait-B	Kuwait-P	Chechens	Mbuti_Pygmies	-0.012925	0.000141	-91.722
Kuwait-B	Kuwait-P	Abkhasians	Mbuti_Pygmies	-0.012901	0.000141	-91.771
Kuwait-B	Kuwait-P	Balkars	Mbuti_Pygmies	-0.012757	0.000139	-91.999
Kuwait-B	Kuwait-P	Balochi	Mbuti_Pygmies	-0.012268	0.000133	-92.002
Kuwait-B	Kuwait-P	Kumyks	Mbuti_Pygmies	-0.012787	0.000138	-92.381
Kuwait-B	Kuwait-P	Ror	Mbuti_Pygmies	-0.0126	0.000136	-92.446
Kuwait-B	Kuwait-P	Lezgins	Mbuti_Pygmies	-0.012976	0.00014	-92.468
Kuwait-B	Kuwait-P	Armenians	Mbuti_Pygmies	-0.012648	0.000137	-92.55
Kuwait-B	Kuwait-P	Pathan	Mbuti_Pygmies	-0.012552	0.000135	-92.939
Kuwait-B	Kuwait-P	Pamiris	Mbuti_Pygmies	-0.012793	0.000138	-93.036

Supplementary Table 3. f_4-statistics for relative allele sharing of the Kuwait population subgroups to ancient West Eurasian individuals.

Pop1	Pop2	Pop3	Pop4	D val	std err	Z val
Kuwait-B	Kuwait-P	Iran_HotuIIIb	Mbuti_Pygmies	-0.012446	0.000298	-41.718
Kuwait-B	Kuwait-P	Natufian	Mbuti_Pygmies	-0.009737	0.000185	-52.651
Kuwait-B	Kuwait-P	Ust_Ishim	Mbuti_Pygmies	-0.009782	0.000178	-54.863
Kuwait-B	Kuwait-P	Iberia_BA	Mbuti_Pygmies	-0.012631	0.000226	-55.838
Kuwait-B	Kuwait-P	Kostenki14	Mbuti_Pygmies	-0.011138	0.000183	-60.786
Kuwait-B	Kuwait-P	Levant_N	Mbuti_Pygmies	-0.011009	0.00017	-64.813
Kuwait-B	Kuwait-P	Anatolia_ChL	Mbuti_Pygmies	-0.012736	0.000196	-64.867
Kuwait-B	Kuwait-P	MA1	Mbuti_Pygmies	-0.012685	0.000188	-67.412
Kuwait-B	Kuwait-P	Clovis_Anzick	Mbuti_Pygmies	-0.011746	0.000174	-67.692
Kuwait-B	Kuwait-P	Switzerland_HG	Mbuti_Pygmies	-0.01231	0.00018	-68.305
Kuwait-B	Kuwait-P	Anatolia_Ottoman	Mbuti_Pygmies	-0.011998	0.000175	-68.744
Kuwait-B	Kuwait-P	Anatolia_IA	Mbuti_Pygmies	-0.012334	0.000178	-69.484
Kuwait-B	Kuwait-P	Iran_recent	Mbuti_Pygmies	-0.012641	0.000181	-69.705
Kuwait-B	Kuwait-P	Steppe_IA	Mbuti_Pygmies	-0.012901	0.000182	-70.812
Kuwait-B	Kuwait-P	Yamnaya_EBA	Mbuti_Pygmies	-0.013176	0.000185	-71.137
Kuwait-B	Kuwait-P	Turkmenistan_IA	Mbuti_Pygmies	-0.012874	0.000176	-73.073
Kuwait-B	Kuwait-P	Levant_BA	Mbuti_Pygmies	-0.011464	0.000156	-73.327
Kuwait-B	Kuwait-P	Iran_N	Mbuti_Pygmies	-0.012524	0.000171	-73.413
Kuwait-B	Kuwait-P	WestSiberia_HG	Mbuti_Pygmies	-0.013052	0.000174	-74.803
Kuwait-B	Kuwait-P	Indus_Diaspora	Mbuti_Pygmies	-0.012226	0.000159	-76.757
Kuwait-B	Kuwait-P	EHG	Mbuti_Pygmies	-0.013078	0.000169	-77.256
Kuwait-B	Kuwait-P	WHG	Mbuti_Pygmies	-0.012308	0.000159	-77.528
Kuwait-B	Kuwait-P	Namazga_CA	Mbuti_Pygmies	-0.012792	0.000162	-79.196
Kuwait-B	Kuwait-P	CHG	Mbuti_Pygmies	-0.012969	0.000161	-80.477
Kuwait-B	Kuwait-P	SHG	Mbuti_Pygmies	-0.012701	0.000157	-80.681
Kuwait-B	Kuwait-P	Anatolia_EBA	Mbuti_Pygmies	-0.012219	0.00015	-81.311
Kuwait-B	Kuwait-P	Armenia_EBA	Mbuti_Pygmies	-0.012849	0.000157	-82.024
Kuwait-B	Kuwait-P	Steppe_LBA	Mbuti_Pygmies	-0.012714	0.000154	-82.311
Kuwait-B	Kuwait-P	Armenia_ChL	Mbuti_Pygmies	-0.012733	0.000154	-82.448
Kuwait-B	Kuwait-P	IranTuran_N	Mbuti_Pygmies	-0.012839	0.000153	-84.031
Kuwait-B	Kuwait-P	Anatolia_MLBA	Mbuti_Pygmies	-0.012436	0.000148	-84.035
Kuwait-B	Kuwait-P	Anatolia_N	Mbuti_Pygmies	-0.012278	0.000145	-84.744
Kuwait-B	Kuwait-P	Iran_ChL	Mbuti_Pygmies	-0.01262	0.000146	-86.155
Kuwait-B	Kuwait-P	Europe_EN	Mbuti_Pygmies	-0.012355	0.000143	-86.614
Kuwait-B	Kuwait-P	IranTuran_BA	Mbuti_Pygmies	-0.013005	0.000145	-89.681
Kuwait-B	Kuwait-P	SouthAsia_H	Mbuti_Pygmies	-0.012351	0.000136	-90.567
Kuwait-B	Kuwait-P	Europe_LNBA	Mbuti_Pygmies	-0.012855	0.000139	-92.308
Kuwait-B	Kuwait-P	Steppe_MLBA	Mbuti_Pygmies	-0.012957	0.00014	-92.503
Kuwait-B	Kuwait-P	Steppe_EMBA	Mbuti_Pygmies	-0.013235	0.000143	-92.656
Kuwait-B	Kuwait-P	SPGT	Mbuti_Pygmies	-0.012574	0.000134	-93.782

Pop1	Pop2	Pop3	Pop4	D val	std err	Z val
Kuwait-B	Kuwait-P	BMAC	Mbuti_Pygmies	-0.012894	0.000136	-94.727
Kuwait-B	Kuwait-S	Iran_HotuIIIb	Mbuti_Pygmies	-0.010315	0.00031	-33.271
Kuwait-B	Kuwait-S	Ust_Ishim	Mbuti_Pygmies	-0.008655	0.00017	-50.929
Kuwait-B	Kuwait-S	Iberia_BA	Mbuti_Pygmies	-0.01201	0.000225	-53.48
Kuwait-B	Kuwait-S	MA1	Mbuti_Pygmies	-0.010145	0.000188	-54.049
Kuwait-B	Kuwait-S	Kostenki14	Mbuti_Pygmies	-0.010133	0.000175	-57.824
Kuwait-B	Kuwait-S	Clovis_Anzick	Mbuti_Pygmies	-0.009666	0.000166	-58.286
Kuwait-B	Kuwait-S	Anatolia_ChL	Mbuti_Pygmies	-0.011936	0.000198	-60.167
Kuwait-B	Kuwait-S	Anatolia_Ottoman	Mbuti_Pygmies	-0.010556	0.000175	-60.244
Kuwait-B	Kuwait-S	Iran_N	Mbuti_Pygmies	-0.01013	0.000167	-60.729
Kuwait-B	Kuwait-S	Natufian	Mbuti_Pygmies	-0.011771	0.000194	-60.782
Kuwait-B	Kuwait-S	WestSiberia_HG	Mbuti_Pygmies	-0.010348	0.000165	-62.533
Kuwait-B	Kuwait-S	Switzerland_HG	Mbuti_Pygmies	-0.011098	0.000174	-63.669
Kuwait-B	Kuwait-S	Yamnaya_EBA	Mbuti_Pygmies	-0.011056	0.000173	-63.784
Kuwait-B	Kuwait-S	Iran_recent	Mbuti_Pygmies	-0.011542	0.000181	-63.865
Kuwait-B	Kuwait-S	Steppe_IA	Mbuti_Pygmies	-0.011064	0.000173	-63.952
Kuwait-B	Kuwait-S	Indus_Diaspora	Mbuti_Pygmies	-0.009857	0.000154	-63.976
Kuwait-B	Kuwait-S	Anatolia_IA	Mbuti_Pygmies	-0.01143	0.000177	-64.575
Kuwait-B	Kuwait-S	EHG	Mbuti_Pygmies	-0.010785	0.000163	-66.18
Kuwait-B	Kuwait-S	Turkmenistan_IA	Mbuti_Pygmies	-0.011062	0.000166	-66.652
Kuwait-B	Kuwait-S	IranTuran_N	Mbuti_Pygmies	-0.010278	0.000149	-68.811
Kuwait-B	Kuwait-S	CHG	Mbuti_Pygmies	-0.010743	0.000155	-69.093
Kuwait-B	Kuwait-S	Namazga_CA	Mbuti_Pygmies	-0.010547	0.000152	-69.43
Kuwait-B	Kuwait-S	SHG	Mbuti_Pygmies	-0.011086	0.000155	-71.497
Kuwait-B	Kuwait-S	Levant_N	Mbuti_Pygmies	-0.012051	0.000166	-72.791
Kuwait-B	Kuwait-S	WHG	Mbuti_Pygmies	-0.011186	0.000152	-73.634
Kuwait-B	Kuwait-S	Steppe_LBA	Mbuti_Pygmies	-0.010802	0.000146	-74.236
Kuwait-B	Kuwait-S	Armenia_ChL	Mbuti_Pygmies	-0.011618	0.000153	-76.005
Kuwait-B	Kuwait-S	Armenia_EBA	Mbuti_Pygmies	-0.011519	0.00015	-76.776
Kuwait-B	Kuwait-S	Levant_BA	Mbuti_Pygmies	-0.012209	0.000159	-76.866
Kuwait-B	Kuwait-S	IranTuran_BA	Mbuti_Pygmies	-0.010751	0.00014	-77.054
Kuwait-B	Kuwait-S	SouthAsia_H	Mbuti_Pygmies	-0.010246	0.00013	-78.556
Kuwait-B	Kuwait-S	Anatolia_EBA	Mbuti_Pygmies	-0.011735	0.000149	-78.749
Kuwait-B	Kuwait-S	Iran_ChL	Mbuti_Pygmies	-0.011279	0.000143	-79.135
Kuwait-B	Kuwait-S	Anatolia_MLBA	Mbuti_Pygmies	-0.011904	0.000147	-80.929
Kuwait-B	Kuwait-S	SPGT	Mbuti_Pygmies	-0.010397	0.000128	-81.142
Kuwait-B	Kuwait-S	Steppe_EMBA	Mbuti_Pygmies	-0.011055	0.000133	-82.933
Kuwait-B	Kuwait-S	Steppe_MLBA	Mbuti_Pygmies	-0.011265	0.000133	-84.545
Kuwait-B	Kuwait-S	BMAC	Mbuti_Pygmies	-0.010909	0.000128	-85.343
Kuwait-B	Kuwait-S	Europe_LNBA	Mbuti_Pygmies	-0.011533	0.000134	-85.785
Kuwait-B	Kuwait-S	Anatolia_N	Mbuti_Pygmies	-0.012307	0.000141	-87.182
Kuwait-B	Kuwait-S	Europe_EN	Mbuti_Pygmies	-0.012301	0.00014	-87.6
Kuwait-P	Kuwait-S	SPGT	Mbuti_Pygmies	0.002178	0.000069	31.616

Pop1	Pop2	Pop3	Pop4	D val	std err	Z val
Kuwait-P	Kuwait-S	SouthAsia_H	Mbuti_Pygmies	0.002105	0.00007	29.992
Kuwait-P	Kuwait-S	Steppe_EMBA	Mbuti_Pygmies	0.00218	0.000075	29.145
Kuwait-P	Kuwait-S	IranTuran_BA	Mbuti_Pygmies	0.002254	0.00008	28.318
Kuwait-P	Kuwait-S	BMAC	Mbuti_Pygmies	0.001985	0.00007	28.159
Kuwait-P	Kuwait-S	IranTuran_N	Mbuti_Pygmies	0.00256	0.000095	26.937
Kuwait-P	Kuwait-S	Indus_Diaspora	Mbuti_Pygmies	0.002369	0.000089	26.628
Kuwait-P	Kuwait-S	WestSiberia_HG	Mbuti_Pygmies	0.002704	0.000103	26.236
Kuwait-P	Kuwait-S	Namazga_CA	Mbuti_Pygmies	0.002245	0.00009	25.018
Kuwait-P	Kuwait-S	Steppe_MLBA	Mbuti_Pygmies	0.001692	0.000072	23.454
Kuwait-P	Kuwait-S	CHG	Mbuti_Pygmies	0.002226	0.000096	23.284
Kuwait-P	Kuwait-S	Steppe_LBA	Mbuti_Pygmies	0.001912	0.000083	23.109
Kuwait-P	Kuwait-S	Iran_N	Mbuti_Pygmies	0.002393	0.000105	22.777
Kuwait-P	Kuwait-S	EHG	Mbuti_Pygmies	0.002293	0.000103	22.195
Kuwait-P	Kuwait-S	MA1	Mbuti_Pygmies	0.00254	0.000118	21.499
Kuwait-P	Kuwait-S	Clovis_Anzick	Mbuti_Pygmies	0.00208	0.000104	19.986
Kuwait-P	Kuwait-S	Yamnaya_EBA	Mbuti_Pygmies	0.00212	0.000112	19.008
Kuwait-P	Kuwait-S	Europe_LNBA	Mbuti_Pygmies	0.001322	0.000073	18.046
Kuwait-P	Kuwait-S	SHG	Mbuti_Pygmies	0.001616	0.000091	17.773
Kuwait-P	Kuwait-S	Steppe_IA	Mbuti_Pygmies	0.001838	0.000106	17.397
Kuwait-P	Kuwait-S	Turkmenistan_IA	Mbuti_Pygmies	0.001812	0.000112	16.204
Kuwait-P	Kuwait-S	Iran_ChL	Mbuti_Pygmies	0.001341	0.000086	15.528
Kuwait-P	Kuwait-S	Armenia_EBA	Mbuti_Pygmies	0.00133	0.000089	14.906
Kuwait-P	Kuwait-S	Anatolia_Ottoman	Mbuti_Pygmies	0.001442	0.000105	13.797
Kuwait-P	Kuwait-S	Armenia_ChL	Mbuti_Pygmies	0.001115	0.000086	12.907
Kuwait-P	Kuwait-S	WHG	Mbuti_Pygmies	0.001122	0.000097	11.572
Kuwait-P	Kuwait-S	Ust_Ishim	Mbuti_Pygmies	0.001127	0.000106	10.661
Kuwait-P	Kuwait-S	Switzerland_HG	Mbuti_Pygmies	0.001212	0.000114	10.658
Kuwait-P	Kuwait-S	Iran_HotuIIIb	Mbuti_Pygmies	0.002132	0.000205	10.382
Kuwait-P	Kuwait-S	Iran_recent	Mbuti_Pygmies	0.001099	0.000115	9.591
Kuwait-P	Kuwait-S	Kostenki14	Mbuti_Pygmies	0.001004	0.00011	9.164
Kuwait-P	Kuwait-S	Anatolia_IA	Mbuti_Pygmies	0.000904	0.000109	8.283
Kuwait-P	Kuwait-S	Anatolia_MLBA	Mbuti_Pygmies	0.000532	0.000083	6.436
Kuwait-P	Kuwait-S	Anatolia_ChL	Mbuti_Pygmies	0.000799	0.000125	6.382
Kuwait-P	Kuwait-S	Anatolia_EBA	Mbuti_Pygmies	0.000484	0.000088	5.507
Kuwait-P	Kuwait-S	Iberia_BA	Mbuti_Pygmies	0.000622	0.000142	4.391
Kuwait-P	Kuwait-S	Europe_EN	Mbuti_Pygmies	0.000054	0.000077	0.694
Kuwait-P	Kuwait-S	Anatolia_N	Mbuti_Pygmies	-0.000029	0.000077	-0.374
**Kuwait-P**	**Kuwait-S**	**Levant_BA**	**Mbuti_Pygmies**	**-0.000746**	**0.000096**	**-7.792**
**Kuwait-P**	**Kuwait-S**	**Levant_N**	**Mbuti_Pygmies**	**-0.001042**	**0.000098**	**-10.645**
**Kuwait-P**	**Kuwait-S**	**Natufian**	**Mbuti_Pygmies**	**-0.002034**	**0.000115**	**-17.622**

Supplementary Table 4. f_4-ratio estimates of Neanderthal ancestry in the Kuwait population.

Outgroup	A	B	C	X	α	std err	Z Score
Chimp	Altai	Vindija	Dinka	Kuwait-B	0.004001	0.005513	0.726
Chimp	Altai	Vindija	Dinka	Kuwait-P	0.003977	0.005932	0.67
Chimp	Altai	Vindija	Dinka	Kuwait-S	0.004917	0.005941	0.828

α - alpha refers to the proportion of Neanderthal in each Kuwaiti subgroup.

Supplementary Table 5. Time of admixture estimate using ALDER.

Test Pop	Ref A	Ref B	P-value	2-Ref decay (time in gen)	Z-Score
Kuwait-B	Yoruba	Estonian	5.70E-41	7.92 ± 0.59	13.54
Kuwait-S	Yoruba	Paniya	1.20E-147	14.98 ± 0.58	25.95
Kuwait-P	Yoruba	Paniya	1.40E-68	15.41 ± 0.88	17.6
